# Supplementary material for: Insights into Transcriptomic Differences in Ovaries between Lambs and Adult Sheep after Superovulation Treatment
Source: Animals (Basel). 2023 Feb 14;13(4):665. doi: 10.3390/ani13040665 (PMC9951745; doi:10.3390/ani13040665)
Supplement: Supplementary file 1 [file animals-13-00665-s001.zip › Figure S1.pdf]

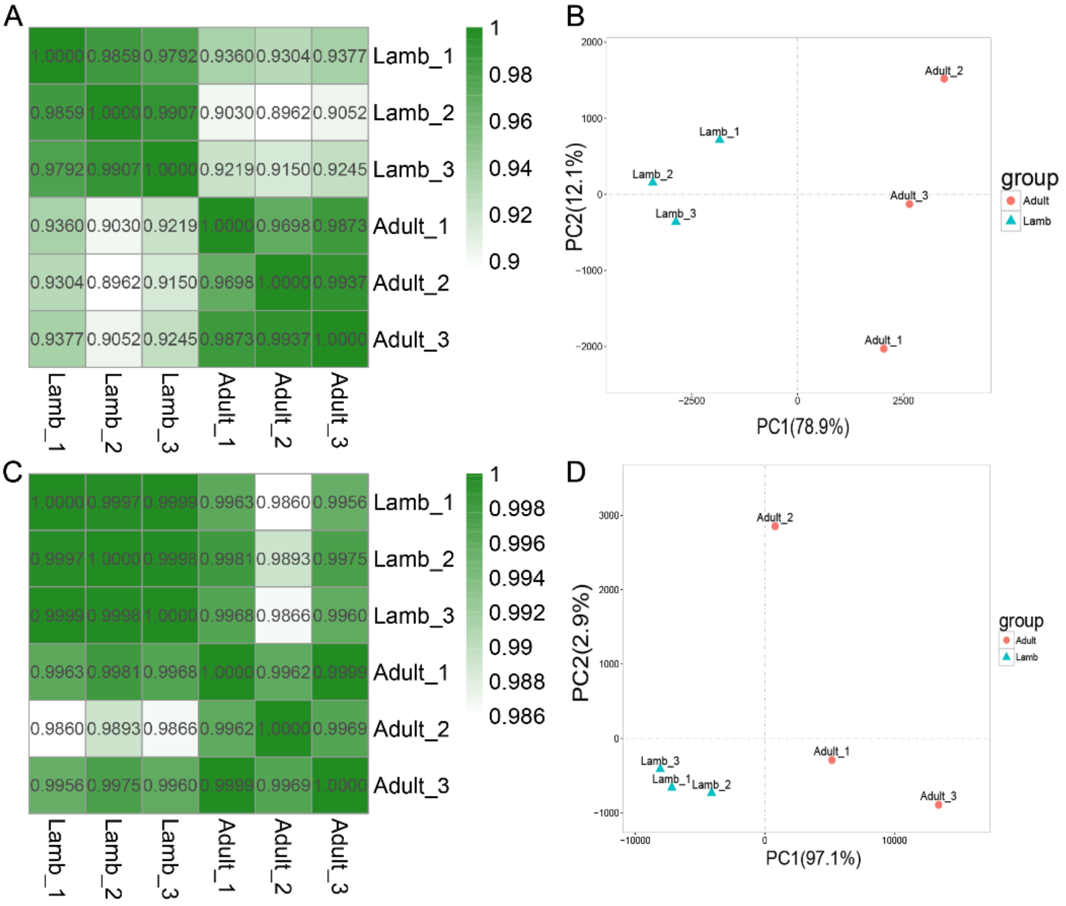

Figure S1. Biological repeats correlation analysis. A) and C) show the sample-to-sample Pearson's correlation results; the darker the cell, the higher correlation of the sample. B) and D) show the PCA results among all the biological repeats.
